# Understanding Acceptance of Genome-Edited Crops and Foods: The Role of Trust, Attitudes, and Perceived Literacy in Italy

**DOI:** 10.3390/foods15061007

**Published:** 2026-03-12

**Authors:** Michele Paleologo, Alessandra Lanubile, Marco Camardo Leggieri, Paolo Gomarasca, Guendalina Graffigna

**Affiliations:** 1 Department of Psychology, Catholic University of the Sacred Heart, L.go Gemelli 1, 20123 Milan, Italy; 2 EngageMinds HUB—Consumer, Food & Health Engagement Research Center, Catholic University of the Sacred Heart, L.go Gemelli 1, 20123 Milan, Italy; 3 Department of Sustainable Crop Production, Catholic University of the Sacred Heart, Via Emilia Parmense 84, 29122 Piacenza, Italymarco.camardoleggieri@unicatt.it (M.C.L.); 4 Department of Philosophy, Catholic University of the Sacred Heart, L.go Gemelli 1, 20123 Milan, Italy; 5 Faculty of Agriculture, Food and Environmental Sciences, Catholic University of the Sacred Heart, Via Bissolati, 74, 26100 Cremona, Italy

**Keywords:** genome editing, food acceptance, trust in science, attitudes, perceived literacy, willingness to buy

## Abstract

Genome-editing (GE) techniques are gaining relevance in the agri-food system for their potential to enhance crop resilience and sustainability, raising questions about consumer acceptance and responsible innovation. Understanding public willingness to buy (WTB) GE foods is therefore essential. While trust in science is often cited as a key driver, its effects are not straightforward. This study examines mechanisms linking trust in science to WTB GE foods, testing the mediating role of attitudes and the moderating role of perceived literacy. A cross-sectional online survey was conducted with a representative sample of Italian adults. Using structural equation modelling, we tested three models: a mediation model, a model including a direct path between trust and WTB, and a moderated model incorporating perceived literacy. Trust predicted more favourable attitudes toward GE, and attitudes were strongly associated with WTB. However, when controlling for attitudes, the direct effect of trust on WTB was negative. Perceived literacy significantly moderated this relationship: higher perceived literacy strengthened the negative trust–WTB association. Overall, generalized trust in science is not sufficient for public acceptance of GE crops and foods. Communication strategies should move beyond trust-building and foster informed, critically engaged consumers.

## 1. Introduction

Recent advances in biotechnology have led to the emergence of New Genomic Techniques (NGTs), a term adopted in line with the current EU regulatory framework to describe a broader category of technologies that includes genome editing (GE), one of the most prominent and publicly debated applications. These include climate change–induced crop stress, such as rising temperatures, water scarcity, and increased pest and disease pressure, as well as the need to accelerate the development of crop varieties with improved resilience and adaptive capacity [1,2]. However, public acceptance of these technologies remains far from granted, as scientific innovation alone does not automatically translate into societal support [3,4]. Research in plant and food biotechnology acceptance has long emphasized the relevance of psychological and communicative dimensions in shaping consumers’ responses to innovation [5].

Beyond individual-level factors, genome editing is embedded within broader governance and policy frameworks aimed at ensuring socially responsible innovation. In the European context, initiatives such as Responsible Research and Innovation (RRI) reflect efforts to align technological development with societal values [6,7]. Nevertheless, public endorsement often remains cautious, highlighting that acceptance depends on more than technological potential alone [8]. Within the acceptance literature, a recent systematic review identified multiple psychosocial drivers of NGTs acceptance—including literacy, trust, attitudes, and value-based orientations—yet empirical findings remain fragmented and sometimes contradictory [9]. In particular, results on the role of generalized trust in science are inconsistent: while some studies report positive associations with GE acceptance [10,11,12], others report weaker or non-significant associations, particularly when trust is measured in a general form or when mediators are not accounted for [13,14,15].

Overall, prior studies indicate that acceptance of biotechnology is shaped by perceived risks and benefits, trust in scientific institutions, and moral concerns; however, the relative weight and interaction of these factors remain debated. These inconsistencies suggest that trust may not act as a simple linear predictor of acceptance, but rather through indirect and conditional mechanisms. Two mechanisms appear particularly relevant. First, attitudes toward GE consistently predict intentions to accept or purchase biotechnology-related products [4]. Second, perceived scientific literacy, defined as individuals’ self-assessed knowledge about scientific issues, may condition how generalized trust in science translates into willingness to buy GE food [16,17,18].

To better understand how these mechanisms operate in practice, it is important to examine them within specific socio-cultural and regulatory contexts. Italy represents a particularly relevant case. The country is characterized by a strong cultural attachment to traditional, high-quality food products [19] and has recently adopted a precautionary stance toward certain food innovations, including the 2023 pre-emptive ban on cultivated meat [20,21]. Such features make Italy a theoretically meaningful context in which to investigate how trust, attitudes, and perceived literacy interact in shaping acceptance of emerging agri-food technologies.

Recent empirical evidence from Italy provides initial insights into consumer perceptions of genome editing and related technologies. Experimental research suggests that information framing alone has limited effects on willingness to pay for genome-edited food products, indicating persistent uncertainty among consumers [22]. Survey-based studies further document low levels of objective knowledge and significant gaps between perceived and actual understanding of new genomic techniques [23]. However, despite these contributions, existing Italian studies have primarily focused on information effects or descriptive patterns of knowledge and attitudes. Little attention has been devoted to formally modelling the psychological mechanisms through which generalized trust in science translates into willingness to buy genome-edited food.

The conceptual framework of this study draws on the Theory of Planned Behaviour [24] and the Elaboration Likelihood Model [25]. In line with the TPB, attitudes toward a behaviour are considered proximal predictors of behavioural intentions. The ELM complements this perspective by suggesting that individuals may rely either on systematic processing of information or on heuristic cues, such as trust, when forming evaluations. Together, these frameworks provide a basis for examining how trust in science shapes attitudes and how perceived literacy may condition the translation of trust into willingness to buy genome-edited foods.

**Figure 1:**
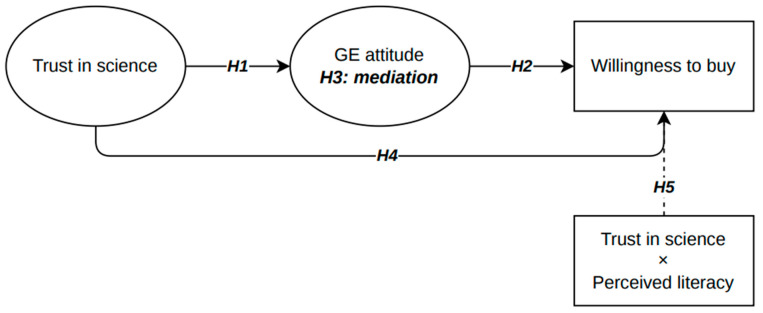
Conceptual model illustrating the hypothesized relationships between trust in science, attitudes toward genome editing (GE), and willingness to buy GE foods.

Based on these premises, the present study aims to investigate the psychosocial mechanisms through which trust in science influences consumer acceptance of crops and food products derived from GE. Specifically, it explores whether this relationship is mediated by attitudes toward GE and whether it is moderated by perceived literacy (see Figure 1). This approach allows us to better understand the cognitive and affective pathways that link general trust in science to concrete behavioural intentions, such as the willingness to buy GE food.

Based on these premises, we developed and tested a structural model linking trust in science, attitudes toward GE, willingness to buy genome-edited foods, and perceived literacy. Specifically, we hypothesized that

**H1.**  *Trust in science is positively associated with favourable attitudes toward GE*.

From the TPB perspective, trust in science may shape evaluative beliefs about emerging technologies, thereby influencing attitudinal orientations toward GE.

**H2.**  *Attitudes toward GE are positively associated with willingness to buy genome-edited food*.

In line with the Theory of Planned Behaviour, attitudes are considered proximal predictors of behavioural intentions.

**H3.**  *Attitudes mediate the effect of trust in science on willingness to buy*.

Integrating TPB and ELM models, trust in science may influence behavioural intentions indirectly through its impact on evaluative attitudes toward GE.

**H4.**  *Trust in science also has a direct effect on willingness to buy, beyond its indirect effect*.

According to ELM, trust may function as a heuristic cue influencing behavioural intentions more directly.

**H5.**  *Perceived literacy moderates the relationship between trust and willingness to buy, such that the strength and direction of the association vary depending on literacy levels*.

Drawing on the Elaboration Likelihood Model, individuals’ perceived level of knowledge may condition the extent to which trust functions as a heuristic cue or is integrated into more systematic evaluations.

## 2. Methods

### 2.1. Participants and Data Collection

A cross-sectional online survey was conducted in June 2024 using a Computer-Assisted Web Interviewing (CAWI) methodology. Participants were recruited through a professional panel provider (SWG), using stratified quota sampling to approximate the Italian adult population in terms of gender, age, geographical area and education. A total of 1000 Italian adults (aged 18 and above) completed the survey. For the present analyses, cases were excluded due to missing responses on key model variables. Comparisons between included (n = 815) and excluded respondents (n = 185) showed no systematic differences across major sociodemographic characteristics (age, education, and geographical area). Participants were informed about the general aims of the study and provided informed consent before accessing the questionnaire. The English translation of the survey instrument is provided in the Supplementary Materials.

Sociodemographic characteristics of the sample are described in Section 3. Prior to model estimation, we assessed the main assumptions for Structural Equation Modelling (SEM) analysis, including multivariate normality, multicollinearity, and the presence of multivariate outliers. Multivariate normality was tested using the Henze–Zirkler test (HZ), multicollinearity was assessed through Variance Inflation Factor (VIF), and multivariate outliers were identified using Mahalanobis distance, with a cutoff corresponding to the 97.5th percentile of the chi-square distribution. Based on these assessments, Maximum Likelihood estimation with robust standard errors (MLR) was used to account for slight deviations from normality.

### 2.2. Measures and Constructs

All constructs were assessed using either multi-item or single-item measures. Validated scales were employed when available, while ad hoc items were developed for constructs not previously covered in the literature. Unless otherwise specified, responses were recorded using 5 or 7 point Likert-type scales, consistent with the original instrument formats. Confirmatory factor analyses (CFAs) were conducted to evaluate the psychometric adequacy of the latent constructs prior to structural modelling.

For the trust in science construct, we relied on the validated 14-item short form [26]. Items with low standardized factor loadings (<0.40) were excluded to ensure factorial validity, resulting in a unidimensional structure with improved model fit. For the Attitude toward GE construct, although it was developed specifically for the present study, item selection and dimensionality were theory-driven and tested through CFA to identify the most robust indicators. Items that did not meet conventional psychometric criteria were excluded.

To ensure comparability across indicators and reduce scale-related variance, all observed variables were standardized prior to model estimation. For latent constructs, standardization was applied at the level of observed indicators, before estimating factor scores or computing interaction terms. All items were administered in Italian and later translated into English for reporting purposes.

#### 2.2.1. Trust in Science

Trust in science was measured using a 14-item version of the Trust in science and Scientists Inventory, originally developed by Nadelson et al. [27]. This shorter version was proposed and validated by Plohl and Musil [26], demonstrates strong internal consistency (α = 0.81) and high correlation with the original 21-item scale (r = 0.97, *p* < 0.001). The items assess trust in scientists’ ethics, integrity, and the reliability of the scientific method (e.g., “We should trust scientists to share their discoveries even if they do not support their own ideas”). Responses were given on a 7-point Likert scale. Reverse-coded items were appropriately recoded prior to analysis. A latent construct was estimated from the 14-item version, and item selection for the final measurement model was guided by CFA results, including standardized loadings and overall model fit indices.

#### 2.2.2. Attitudes Toward Genome Editing

Attitudes toward GE technology were assessed using seven items developed for the purpose of this study, based on previous literature on emerging plant and food biotechnologies. Items reflected both perceived benefits and ethical or environmental concerns (e.g., “Genome editing has the potential to improve the nutritional value of food”; “Using genome editing techniques means interfering with nature”). Responses were provided on a 5-point scale. A latent factor was constructed using the items that showed adequate factor loadings and internal consistency.

#### 2.2.3. Willingness to Buy (WTB)

Willingness to buy foods obtained through GE was measured using a single item: “Would you buy foods knowing they are obtained through genome editing techniques?” Participants responded on a 5-point scale. The item was reverse-coded so that higher values indicated greater willingness to buy. The variable was standardized prior to model estimation.

#### 2.2.4. Perceived Literacy

Perceived literacy was measured with a single item: “How informed do you consider yourself about genome editing of food and organisms?” Participants responded on a 5-point scale (1 = Very informed; 5 = Not informed at all), which was reverse-coded and z-standardized so that higher scores reflected higher perceived literacy.

### 2.3. Analytical Strategy

All analyses were conducted in R (version 4.2.0) using the Lavaan package (version 0.6–12), visualization of moderation effects was performed using the interactions package, and SEM diagrams were generated using LavaanPlot. Structural Equation Models (SEMs) were estimated using MLR.

Three structural models were estimated to test the hypothesized relationships:

1. A full model including both direct and indirect effects of trust on willingness to buy (WTB);
2. A mediation-only model, excluding the direct path;
3. A moderated model with an interaction term between trust and perceived literacy predicting WTB.

Observed variables were standardized before inclusion in the models. To test moderation, an interaction term was computed by multiplying the standardized scores of trust in science and perceived literacy. For latent constructs, only items with standardized factor loadings above 0.40 were retained, in order to ensure model parsimony and adequate fit. Model fit was evaluated using the following indices:

- Chi-square (χ^2^)
- Comparative Fit Index (CFI)
- Tucker–Lewis Index (TLI)
- Root Mean Square Error of Approximation (RMSEA) and its 90% confidence interval
- Standardized Root Mean Square Residual (SRMR)

To assess the internal consistency and convergent validity of the latent constructs, we computed Composite Reliability (CR) and Average Variance Extracted (AVE), based on standardized factor loadings obtained from the confirmatory factor analysis. Discriminant validity was assessed using the Fornell–Larcker criterion, comparing the square root of AVE for each construct with their mutual correlation.

To assess whether the direct effect of trust on willingness to buy significantly improved model fit, we performed a chi-square difference test comparing the full model (Model 1) with the mediation-only model (Model 2).

## 3. Results

### 3.1. Sample Description

The final sample consisted of 815 Italian respondents aged 18 years or older. Gender distribution was balanced, with 45.6% men, 54.1% women, and 0.3% identifying as non-binary. Age was broadly distributed across three categories: 16.3% were aged 18–34, 30.3% 35–54, 53.4% were 55 years and older. Regarding educational level, 10.9% of respondents had lower secondary education or less, 54.8% had completed or were currently attending upper secondary education, and 34.3% were either enrolled in or had completed tertiary education. A detailed breakdown is provided in Table 1.

### 3.2. Measurement Model

To evaluate the psychometric properties of the latent constructs, CFAs were conducted. For trust in science, an initial CFA tested the full 14-item scale [26]. Items with standardized loadings below 0.40 were excluded, resulting in a refined 9-item model with loadings ranging from 0.64 to 0.84. The model showed good fit: χ^2^ (27) = 171.98, *p* < 0.001; CFI = 0.973; TLI = 0.958; RMSEA = 0.061 [0.053, 0.070]; SRMR = 0.025.

For Attitudes toward GE, a CFA was performed on a 7-item scale developed for this study. Two items were excluded due to low or cross loadings. The final 5-item solution showed acceptable fit: χ^2^ (5) = 33.63, *p* < 0.001; CFI = 0.987; TLI = 0.975; RMSEA = 0.051 [0.036, 0.068]; SRMR = 0.019. Standardized loadings ranged from 0.60 to 0.81.

Both constructs demonstrated good psychometric properties: convergent validity (AVE = 0.543 for trust in science, 0.516 for GE Attitude), composite reliability (CR = 0.853 and 0.733, respectively), and internal consistency (Cronbach’s alpha > 0.70). Fornell–Larcker criteria for discriminant validity were also met, with the square roots of AVE exceeding the latent correlation (r = 0.583). A complete table reporting all standardized loadings, squared loadings, AVE and CR values for each construct is available in Appendix A. In practical terms, these results indicate that the selected items reliably captured the intended constructs and that trust in science and attitudes toward GE represent empirically distinct yet related dimensions.

Discriminant validity was also supported according to the Fornell–Larcker criterion: the square roots of the AVE for both trust in science (√AVE = 0.737) and GE Attitude (√AVE = 0.718) were greater than their latent correlation (r = 0.583), indicating that each construct shared more variance with its own indicators than with the other latent construct.

### 3.3. Structural Models

#### 3.3.1. Assumption Check

Results from the Henze–Zirkler test indicated a significant deviation from multivariate normality across the 15 items used in the measurement model (HZ = 2.03, *p* < 0.001). Accordingly, robust estimation methods were used to account for non-normality in the structural equation modelling (MLR estimator in lavaan). This means that the model parameters were estimated using procedures that correct for deviations from normality, increasing the robustness of the results.

Multicollinearity among predictors in the moderated model was assessed using VIF. All VIF values were well below the conventional threshold of 5 (VIFs < 1.1), indicating no concerns about collinearity between the predictor (Trust), the moderator (Perceived Literacy), and their interaction term. This suggests that the predictors included in the model did not overlap excessively and could be interpreted independently.

Multivariate outliers were assessed via Mahalanobis distance using Trust in science and Perceived Literacy as predictors. No cases exceeded the critical cutoff for *p* < 0.001 (χ^2^ (2) = 13.82), indicating the absence of influential multivariate outliers. Therefore, no extreme response patterns were identified that could distort the structural relationships.

#### 3.3.2. Model 1—Full Mediation Model with Direct Path

**Figure 2:**
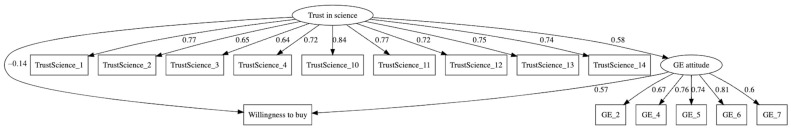
Structural equation model showing the measurement and structural components of the mediation model. Latent constructs are represented by ellipses and observed variables by rectangles. Values reported on the arrows indicate standardized factor loadings and standardized path coefficients.

The full model, including both direct and indirect paths from Trust to WTB, demonstrated good fit: χ^2^ (88) = 371.77, *p* < 0.001; CFI = 0.953; TLI = 0.944; RMSEA = 0.063 [0.056, 0.070]; SRMR = 0.050 (see Figure 2 for the path diagram).

Standardized path coefficients:

- Trust → Attitudes: β = 0.583, *p* < 0.001
- Attitudes → WTB: β = 0.575, *p* < 0.001
- Trust → WTB: β = −0.137, *p* = 0.001
- Together, these coefficients indicate that trust in science strongly predicts more favourable attitudes, which in turn are associated with higher willingness to buy GE foods.

Interestingly, the direct path from trust to willingness to buy was negative, despite the overall positive indirect effect via attitudes. This indicates that, once attitudes are accounted for, higher trust in science is associated with lower willingness to buy GE foods. The model explained R^2^ = 0.339 for attitudes toward GE and R^2^ = 0.257 for willingness to buy GE foods, indicating that approximately one quarter of the variance in willingness to buy was accounted for by the model.

#### 3.3.3. Model 2—Mediation-Only Model

In the model where the direct path from Trust to WTB was constrained to zero, the model fit remained acceptable: χ^2^ (89) = 382.02, CFI = 0.951, TLI = 0.943, RMSEA = 0.064, SRMR = 0.053.

- Trust → Attitudes: β = 0.572, *p* < 0.001
- Attitudes → WTB: β = 0.484, *p* < 0.001

The model explained R^2^ = 0.328 for attitudes toward GE and R^2^ = 0.234 for willingness to buy GE foods.

A chi-square difference test using the Satorra-Bentler scaled correction indicated that the full model (Model 1) provided a significantly better fit than the mediation-only model (Model 2): Δχ^2^ (1) = 10.25, *p* = 0.001. This supports the inclusion of the direct path from Trust to WTB, despite its negative coefficient.

Overall, these findings reinforce the mediating role of attitudes in the relationship between trust and behavioural intention. This comparison confirms that including the direct path provides a more accurate representation of the relationship between trust and willingness to buy.

#### 3.3.4. Model 3—Moderated Model

When perceived literacy was introduced as a moderator of the relationship between trust and WTB (via an interaction term), model fit remained adequate though slightly reduced: χ^2^ (102) = 509.27; CFI = 0.934; TLI = 0.923; RMSEA = 0.070 [0.064, 0.076]; SRMR = 0.098.

Key coefficients:

- Interaction Trust × Literacy → WTB: β = −0.224, *p* < 0.001
- Trust → WTB: β = −0.08, *p* = 0.043
- Attitudes → WTB: β = 0.600, *p* < 0.001

**Figure 3:**
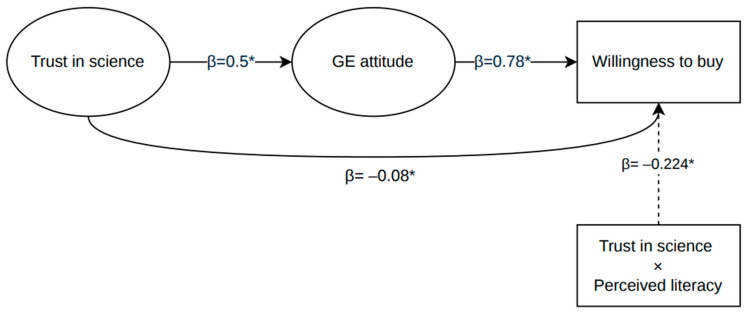
Standardized path coefficients of the moderated mediation model. Values indicate standardized coefficients; * *p* < 0.05.

The model explained R^2^ = 0.340 for attitudes toward GE and R^2^ = 0.360 for willingness to buy GE foods (see Figure 3).

The significant interaction suggests that perceived literacy moderates the effect of trust on willingness to buy. Specifically, higher levels of perceived literacy amplify the negative effect of trust in science on purchase intention, indicating that individuals who perceive themselves as more informed may engage more critically with scientific narratives, particularly in the context of GE food. In practical terms, the relationship between trust and willingness to buy varies depending on how knowledgeable individuals perceive themselves to be.

## 4. Discussion

The present study aimed to explore the relationship between trust in science and consumers’ willingness to buy crops and foods obtained through GE, considering the mediating role of attitudes and the moderating role of perceived literacy. Our findings provide nuanced insights into these dynamics and contribute to the broader debate on how psychosocial variables shape public acceptance of GE technology. The positive association between trust in science and attitudes toward GE is consistent with prior studies reporting that higher institutional trust is associated with more favourable evaluations of emerging biotechnologies [10,11,12]. Similarly, the strong link between attitudes and willingness to buy aligns with established findings within the Theory of Planned Behaviour framework, where attitudes represent a proximal determinant of behavioural intentions [24]. However, the negative direct effect of trust on willingness to buy, once attitudes are controlled for, helps clarify the inconsistent results reported in previous research on generalized trust in science [10,11,12,13,14,15], suggesting that its influence may operate through more complex and conditional mechanisms.

Second, attitudes toward GE were strongly associated with willingness to buy GE foods, supporting a well-established pathway in the literature on crops and food biotechnology acceptance [4]. The strength of this relationship underlines the importance of targeting attitudes in communication strategies aimed at promoting informed acceptance of GE technology.

The indirect effect of trust on WTB, mediated by attitudes, was significant and positive, supporting our third hypothesis. However, the inclusion of a direct path from trust to WTB revealed an unexpected result: when attitudes are controlled for, higher trust in science is associated with lower willingness to buy. This counterintuitive result may suggest the presence of underlying ambivalences in how people conceptualize scientific trust possibly linked to broader cultural or ideological orientations, or to conditional trust contingent on institutional transparency and perceived benefit sharing.

This unexpected negative association has also been observed in previous research. Several studies have shown that general trust in science does not always lead to higher levels of acceptance of specific technologies, especially in the domain of food and biotechnology. For instance, Bearth et al. [14] and Yang and Hobbs [15] highlight that the predictive power of trust can diminish or even reverse when public concerns about naturalness, ethics, or corporate interests come into play. Baum et al. [13] similarly discuss how trust in science may coexist with scepticism toward how technologies are implemented or regulated. U.S. public opinion clearly distinguishes the safety of gene editing in the medical field from that in agriculture, demanding more robust evidence for the latter [28]. These findings support the idea that trust can be conditional not just on the perceived credibility of scientists but also on the transparency and perceived fairness of the broader innovation ecosystem.

Furthermore, this ambivalence mirrors the tone of many public and media narratives around GE. Kossmann and colleagues [29] analyzed German press coverage of CRISPR/Cas9 from 2012 to 2023 and found that media framing oscillated between discourse on human health and agricultural/food applications, with positive frames (innovation, productivity) dominating but critical frames (ecology, public opposition) remaining present especially in relation to NGTs applied to agriculture and food production. In parallel, the Annenberg Science Media Monitor (mid-2012 to mid-2017) reports that in headlines linking CRISPR to ethical issues, 38% were framed positively, 43% neutrally, and 19% negatively, reflecting recurrent oscillations between hope and concern in media discourse [30]. Though not plant/animal-specific, this dataset provides robust evidence of fragmented and ambivalent framing around CRISPR in general.

Taken together, these findings support the argument that even individuals with high generalized trust in science may exhibit resistance to specific applications, particularly those involving food or agricultural technologies, when media narratives invoke issues of ethics, naturalness, corporate control, or transparency.

In the Italian context, such ambivalence may be particularly pronounced. Italy is characterized by a strong cultural attachment to traditional, high-quality food products and by recurrent public debates around food sovereignty, naturalness, and territorial identity. The 2023 pre-emptive ban on cultivated meat further illustrates a precautionary institutional stance toward certain food innovations. Within such a socio-regulatory environment, generalized trust in science may coexist with heightened sensitivity toward applications perceived as intervening in the “natural” domain of food. This context may help explain why trust does not translate linearly into willingness to buy, and why attitudes play a central mediating role. At the same time, this suggests that the psychosocial mechanisms observed here should be interpreted within their cultural and regulatory setting, and may operate differently in countries where NGT products are already commercialized and normalized.

These dynamics may also reflect a deeper issue related to how the concept of science itself is socially represented. Trust in science, as measured by the scale developed by Plohl & Musil [26], may not capture the nuances of individual conceptualizations of what “counts” as science in different domains (e.g., GE). People may express generalized trust while holding divergent views on what science should be, depending on their moral, political, or epistemological orientations. In the case of GE, symbolic associations with genes as fundamental units of life can trigger emotionally charged and often unconscious reactions, rooted in perceptions of naturalness, purity, and existential control. Therefore, GE might serve as a particularly rich case to explore how public attitudes are shaped not only by informational factors but also by symbolic and moral dimensions. This suggests the need for more refined measures of trust that can better account for such layered meanings, especially when evaluating attitudes toward emerging biotechnologies.

Finally, our moderation analysis indicated that perceived literacy significantly shapes how trust in science translates into behavioural intentions. Contrary to expectations, higher perceived literacy strengthened the negative direct effect of trust in science on willingness to buy genome-edited food. This suggests that individuals who feel more informed may adopt a more critical stance, potentially balancing their general trust in science with a deeper awareness of the specific implications, risks, or ethical concerns related to GE. This pattern may also be explained through the lens of the availability heuristic, whereby individuals judge the likelihood or severity of an event based on how easily examples come to mind [31,32]. Media content that is alarming, emotional, or ethically controversial tends to be more memorable, and thus more influential in shaping risk perceptions even among those with high perceived literacy. In the context of GE, critical narratives or high-profile controversies may be cognitively more available, reinforcing scepticism or hesitation despite general trust in science.

For policymakers, these findings indicate that promoting generalized trust in science alone is insufficient to foster acceptance of genome-edited foods. Communication should move beyond abstract appeals to scientific authority and instead address concrete benefits, risks, and regulatory safeguards. Public engagement initiatives should favour deliberative approaches over one-way information campaigns, particularly for individuals who perceive themselves as knowledgeable and may adopt a more critical stance. Transparency in governance and oversight appears essential to prevent trust from turning into scepticism when specific applications are evaluated.

Overall, our findings confirm and expand previous research by showing that trust in science is a necessary but not sufficient condition for consumer acceptance of GE technology. Its effects depend both on the cognitive-affective filter of attitudes and on the metacognitive dimension of perceived literacy. These insights can inform both theoretical models of science communication and practical strategies to engage the public on sensitive agri-food innovations.

### Limitations

While the present study offers valuable insights into the psychosocial mechanisms underlying public acceptance of GE crops and foods, some limitations must be acknowledged.

First, the cross-sectional design does not allow for causal inferences. Although structural equation modelling enables the testing of theoretically grounded relationships, longitudinal or experimental studies would be needed to confirm the directionality of the effects.

Second, willingness to buy was measured using a single item, which despite being common practice in similar studies may not fully capture the complexity of consumer behaviour. Future research could incorporate behavioural measures or more nuanced items that account for context-specific considerations (e.g., labelling, price, product type). For instance, experimental evidence shows that mandatory bioengineered labels can either raise or dampen consumers’ WTB depending on their information-seeking style [33].

Third, perceived literacy was assessed through a single self-reported item. While this aligns with previous work on subjective knowledge, it does not allow comparison with objective scientific literacy. A combined measure could yield richer insights into the interplay between perceived and actual knowledge.

Fourth, another potential limitation concerns the measurement of trust in science. Although the scale used is validated and widely adopted, it reflects a general conceptualization of scientific trust. This may not fully capture the symbolic, moral, or ideological dimensions that emerge in response to specific technologies such as GE. Future studies should consider the development of more targeted instruments that can account for how individuals conceptualize science and how these representations intersect with political values, moral concerns, and personal beliefs.

Finally, the generalizability of our findings is limited by the use of a single-country sample (Italy). Cultural, institutional, and regulatory factors vary widely across countries and may shape public attitudes and trust dynamics differently. Cross-national studies would be useful to explore these contextual variations. Recent national data from Sweden, for instance, highlight how acceptance rises when the stated purpose is environmental or animal-welfare related [34], underscoring the value of multi-country comparisons.

## 5. Conclusions

This study examined the mechanisms linking trust in science to willingness to buy genome-edited (GE) foods in a representative sample of Italian adults. The findings confirm that trust in science positively predicts favourable attitudes toward GE, and that attitudes are a strong proximal determinant of willingness to buy. The mediation analysis supports the hypothesis that attitudes transmit the effect of trust on behavioural intention. However, when attitudes are controlled for, trust shows a negative direct association with willingness to buy, indicating a more complex and potentially ambivalent role of generalized scientific trust in the context of food biotechnology.

Furthermore, perceived literacy significantly moderates this relationship. Higher perceived literacy strengthens the negative direct association between trust and willingness to buy, suggesting that individuals who perceive themselves as more informed may engage in more critical evaluation processes when considering GE foods.

Overall, the results demonstrate that generalized trust in science is not sufficient to ensure acceptance of GE crops and foods. Public acceptance emerges from the interaction between trust, evaluative attitudes, and perceived knowledge. These findings highlight the need for communication strategies that go beyond trust-building and instead foster informed, transparent, and critically engaged public dialogue around emerging agri-food technologies.

Longitudinal designs would help clarify the temporal ordering between trust, attitudes, and behavioural intentions, allowing a more precise assessment of the dynamic processes suggested by the present findings. Moreover, cross-national comparisons could examine whether the observed moderation by perceived literacy holds in different cultural and regulatory contexts, particularly in countries where NGT products are already commercialized.

## Figures and Tables

**Table 1:** Sociodemographic characteristics of the sample (N = 815).

Variable	Category	%
Gender	Women	54.1
	Man	45.6
Age	18–34	16.3
	35–54	30.3
	Over 55	53.4
Education	Lower secondary or less	10.9
	Upper secondary	54.8
	Tertiary education	34.3

## Data Availability

The data that support the findings of this study are available from the corresponding author upon reasonable request. The data are not publicly available due to privacy and ethical restrictions related to the protection of participants and the conditions of the informed consent.

## Supplementary Materials

The following supporting information can be downloaded at: https://www.mdpi.com/article/10.3390/foods15061007/s1, English translation of the survey instrument.

## Appendix A

The following tables report standardized factor loadings, squared loadings, Average Variance Extracted (AVE), and Composite Reliability (CR) for the two latent constructs included in the SEM analyses: Trust in Science and GE Attitude.

**Table A1:** Standardized factor loadings and squared loadings, for “Trust in Science”.

Item	Std. Loading	Squared Loading
TrustScience_1	0.766	0.587
TrustScience_2	0.650	0.423
TrustScience_3	0.644	0.415
TrustScience_4	0.723	0.523
TrustScience_10	0.843	0.710
TrustScience_11	0.775	0.601
TrustScience_12	0.723	0.523
TrustScience_13	0.745	0.555
TrustScience_14	0.742	0.550

**Table A2:** Average Variance Extracted (AVE), and Composite Reliability (CR) for “Trust in Science”.

Measure	Value
AVE	0.543
CR	0.853

**Table A3:** Standardized factor loadings and squared loadings, for the latent construct “GE Attitude”.

Item	Std. Loading	Squared Loading
GE_2	0.668	0.446
GE_4	0.755	0.570
GE_5	0.741	0.549
GE_6	0.805	0.648
GE_7	0.604	0.365

**Table A4:** Average Variance Extracted (AVE) and Composite Reliability (CR) for “GE Attitude”.

Measure	Value
AVE	0.516
CR	0.733
