# Supplementary material for: Understanding Acceptance of Genome-Edited Crops and Foods: The Role of Trust, Attitudes, and Perceived Literacy in Italy
Source: Foods. 2026 Mar 12;15(6):1007. doi: 10.3390/foods15061007 (PMC13024929; doi:10.3390/foods15061007)
Supplement: Supplementary file 1 [file foods-15-01007-s001.zip › foods-4117161-supplementary.pdf]

---Page start---INTRODUCTION

INTRODUCTION AND PRIVACY

The Catholic University of Milan is conducting the research project " **Communicating Science** ", focusing on people's opinions regarding topics related to science and scientific communication.

In the questionnaire, we will ask you some questions on these topics and more generally about yourself.

We will also provide you with a short scientific text and then ask your opinion about it.

Type: question only

---Page start---

socio_demo) Initial sample profiling (socio-demographic screening)

---Page start---

intro_prof) To begin, we will ask you for some data for statistical purposes.

Type: question only

---Page start---

p_sesso) You are: [SINGLE]

1. Man
2. Woman
3. Non-binary/other

---Page start---

p_data_nascita) What is your date of birth? (dd/mm/yyyy)

Type: date

---Page start---

p_comune_istat) In which municipality do you live? Type: open text

a_istat_reg) Region (Istat) Type: nominal

a_istat_prov) PROVINCE Type: nominal

---Start of page---

p_scolarita) What is your highest educational qualification? [SINGLE]

1) Primary school / no qualification
2) Lower secondary school
3) Attending upper secondary school
4) Vocational/professional qualification (3-5 years)
5) Upper secondary school diploma (5 years)
6) Currently attending university / no degree yet obtained
7) University diploma / short degree
8) Bachelor's degree (first cycle)
9) Master's degree (second cycle) or 4-5 year degree
10) Postgraduate master's / specialist training school
11) PhD

---Start of page---

Let's talk about the validity of scientific discoveries over time.

Which of the following statements do you most agree with?

Rotation type: random

1) All phenomena discovered and confirmed by science will remain true forever.
2) Some phenomena discovered and confirmed by science will remain true forever.
3) Certainly, some phenomena discovered and confirmed by science will be revised in the future.
4) Everything that science currently considers a fact will be revised in the future.
5) Everything that science currently considers a fact is certainly wrong, because science is not able to discover the truth.
6) None of the above [exclusive]
7) I don't know [exclusive]

*** Start of question rotation - Type: random ***
---Start of page---
Let's talk about scientists now.
For each of the following statements, please indicate how much you agree, using a scale from 1 (Completely disagree) to 7 (Completely agree).
Type: encapsulated
Rotation type: random
1) Scientists do not consider evidence that contradicts their work (R)
2) Scientific theories are weak explanations (R).
3) Scientists intentionally keep their research work secret. (R)
4) Scientists do not value other people's ideas. (R)
5) You must trust the work of scientists.
6) You must trust the honesty of scientists in their work.
7) You must trust that scientists respect ethical values in their work.
SCALE
1) 1 = Completely disagree
2) 2 = Disagree
3) 3 = Somewhat disagree
4) 4 = Neither agree nor disagree
5) 5 = Somewhat agree
6) 6 = Agree
7) 7 = Completely agree
---Start of page---
---Inizio pagina---
Let us now talk about genome editing of food and organisms.
In general, how well informed do you feel about genome editing? [SINGLE]
1) Very
2) Quite
3) So-so
4) Not much
5) Not at all
---Start of page---
Below you will find a brief information sheet about genome editing techniques. We ask you to read it carefully. Then click the arrow to continue.
"Genome editing is a technology that makes it possible to modify, replace or introduce DNA sequences with great precision at specific points in the genome.
In the agri-food sector, genome editing is used to develop plants with desirable characteristics, such as greater resistance to diseases or higher yields.
For example, plants can be modified to be more resistant to pests and adverse climatic conditions, or to have greater quantities of nutrients or improved preservation of the foods derived from them."
Type: question only

---Start of page---
Below are some statements made by previous respondents regarding genome editing.
In light of what you know and have read previously, indicate your degree of agreement from 1 (Completely disagree) to 7 (Completely agree)
Type: encapsulated
Rotation type: random
1) Genome editing could create food with higher nutritional values.
2) It is not possible to know whether consuming foods that have undergone genome editing may cause long-term harm.
3) Genome editing can reduce pesticide contamination in the environment.
4) Crops that have undergone genome editing processes are harmful to the environment.
5) Using genome editing techniques means interfering with nature.
6) It is wrong for humans to modify the natural genome of plants through genome editing techniques.
7) Foods derived from genome editing techniques are imposed on developing nations by more developed countries.
SCALE
1) 1 = Completely disagree
2) 2 = Disagree
3) 3 = Fairly disagree

4) 4 = Neither agree nor disagree
5) 5 = Fairly agree
6) 6 = Agree
7) 7 = Completely agree
8) I don't know [exclusive]
---Start of page---
Would you consume foods knowing they are derived from genome editing techniques? [SINGLE]
1) Definitely yes
2) Probably yes
3) Maybe yes, maybe no
4) Probably not
5) Definitely not
---Start of page---
Would you purchase foods knowing they are derived from genome editing techniques? [SINGLE]
1) Definitely yes
2) Probably yes
3) Maybe yes, maybe no
4) Probably not
5) Definitely not

---Start of page--- DEBRIEFING
Thank you for taking part in this research.
Throughout the questionnaire, we asked you a series of general questions to understand your perception of science, as well as your opinion on more specific topics such as genome editing.
If you would like further information about this research, you can contact Università Cattolica by writing to ricerca.comunicarelascienza@unicatt.it
Type: question only
